# Nanoflowers on Microporous Graphene Electrodes as a Highly Sensitive and Low-Cost As(III) Electrochemical Sensor for Water Quality Monitoring

**DOI:** 10.3390/s23063099

**Published:** 2023-03-14

**Authors:** Mahatthanah Kosuvun, Pobporn Danvirutai, Daranee Hormdee, Arnut Chaosakul, Visanu Tanboonchuy, Apirat Siritaratiwat, Sirirat Anutrakulchai, Amod Sharma, Adisorn Tuantranont, Chavis Srichan

**Affiliations:** 1 Faculty of Engineering, Khon Kaen University, Khon Kaen 40002, Thailand; mahatthanah@kkumail.com (M.K.); pobporn.danvirutai@gmail.com (P.D.); darhor@kku.ac.th (D.H.); arnut@kku.ac.th (A.C.); visanu@kku.ac.th (V.T.); apirat@kku.ac.th (A.S.); 2 Research and Development Division, T. Robotics, Co., Ltd., Khon Kaen 40000, Thailand; 3 Research Center for Environmental and Hazardous Substance Management (EHSM), Khon Kaen University, Khon Kaen 40002, Thailand; 4 Faculty of Medicine, Khon Kaen University, Khon Kaen 40002, Thailand; sirirt_a@kku.ac.th (S.A.); amodssharma@gmail.com (A.S.); 5 Chronic Kidney Disease Prevention in the Northeast of Thailand (CKDNET), Khon Kaen University, Khon Kaen 40002, Thailand; 6 Graphene and Printed Electronics for Dual-Use Applications Research Division (GPERD), National Science and Technology Development Agency (NSTDA), Pathum Thani 12120, Thailand; adisorn.tua@nstda.or.th

**Keywords:** arsenic, electrochemical sensing, graphene, nanoflowers, microporous graphene

## Abstract

In this work, we report a low-cost and highly sensitive electrochemical sensor for detecting As(III) in water. The sensor uses a 3D microporous graphene electrode with nanoflowers, which enriches the reactive surface area and thus enhances its sensitivity. The detection range achieved was 1–50 ppb, meeting the US-EPA cutoff criteria of 10 ppb. The sensor works by trapping As(III) ions using the interlayer dipole between Ni and graphene, reducing As(III), and transferring electrons to the nanoflowers. The nanoflowers then exchange charges with the graphene layer, producing a measurable current. Interference by other ions, such as Pb(II) and Cd(II), was found to be negligible. The proposed method has potential for use as a portable field sensor for monitoring water quality to control hazardous As(III) in human life.

## 1. Introduction

Human activities, such as mining, the production of pigments and pesticides, and others, have altered the levels of arsenic (As) in nature. Of importance, arsenic is a hazardous element to human health, and its poisoning causes fatal diseases, including cancer, liver damage, kidney disease, and disturbances of the nervous system [1]. Arsenic toxicity occurs through food and water when metals accumulate in agricultural products and the living environment. According to the World Health Organization (WHO), arsenic levels in drinking water must be lower than 10 ppb [2,3]. Arsenic exposure can have a fatal effect on health; it can cause colon cancer [4], cardiovascular diseases, pulmonary abnormalities, and renal failure [5]. The best way to prevent arsenic poisoning is to avoid arsenic-contaminated water. Therefore, an easy, inexpensive, and accurate method to determine arsenic is inevitable at the current time for better human health.

There are several methods for determining the level of arsenic contamination. High-Performance Liquid Chromatography (HPLC) and Inductively Coupled Plasma Mass Spectrometry (ICP-MS) are the traditional methods, with the limit of detection (LOD) down to microgram levels or less [6]. Likewise, atomic fluorescence spectroscopy [7], gold-nanoparticle colorimetric detection [8,9], and Surface-Enhanced Raman Scattering (SERS) [10] are other candidates that can achieve high precision and sensitivity at the expense of a trade-off. However, these highly expensive methods make regular and near real-time field monitoring of arsenic levels infeasible. The colorimetric method has also been used to determine total arsenic concentration. Although they are easy to use and less expensive than large spectroscopic apparatuses, this method lacks sensitivity (2.0 µgL^−1^ LOD) compared to other methods. They are mainly suitable for the semi-quantitative determination of high concentrations of arsenic in water. In addition, the colorimetric method cannot be used to make a real-time measurement on the AS(III) level, as in chronoamperometry.

Electrochemical methods are notable for arsenic determination. Despite this expense, hybrid gold-platinum nanoparticles with polyaniline have been proposed as an electrode for As(III) detection via anodic stripping voltammetry and achieve 19.7 nM or 1.48 ppb LOD [11]. Reduced graphene oxide (rGO) and flat graphene electrodes have been proposed with LODs of 1.19 and 500 ppb, respectively [12]. A mercaptoethylamine-modified gold electrode has been proposed and achieves a 0.2–300 ppb detection range [13]. A hybrid graphene-platinum (G-Pt) electrode has been reported to achieve ultra-high sensitivity with 0.008 ppb LOD. Hybrid G-Pt shows a magnificently greater sensitivity compared to Au-Pt electrodes. This gives a clue that a graphene-based electrode could possibly be a low-cost, high-performance solution for the As(III) sensor in the absence of expensive Pt and Au. Recent advances in As(III) electrochemical sensing approaches have been summarized in [14]. However, there has been no report of using microporous graphene foam with nanoflowers as an electrode for As(III) sensing, which will be reported in this work.

**Figure 1:**
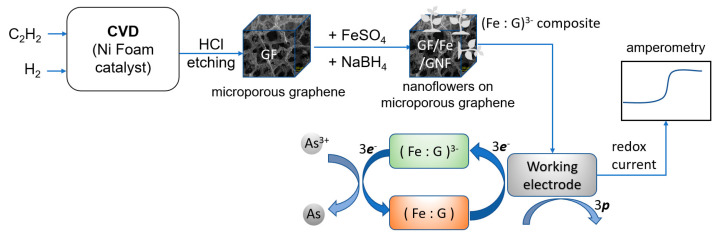
Overall picture of the experiments.

In this work, we aimed to develop a low-cost and highly sensitive electrochemical sensor composed of three-dimensional graphene foam (GF) and composite ferrous-carbon nanoflowers (GNF)—the electrode material was then abbreviated as GF/GNFs—to determine arsenic levels in the water. Earlier, the GF/Ni-based electrochemical sensor was revealed to have ultra-high sensitivity [15,16]. Additionally, a three-dimensional GF with nanoscale decorations has been reported as ideal for the development of several sensors. For instance, GF decorated with AgNPs has been reported to serve as a SERS substrate [17]. Nanoflowers fabricated using metal and organic compounds have been reported as promising structures for a higher sensitivity actuator due to their micro-nano scale increment of reactive surface area [18]. In our case, carbon from graphene foam and Fe/Ni alloys together catalyze the growth of hybrid nanoflowers on graphene/Ni foam. In this work, we reported for the first time the GF/GNFs electrode (GF/GNFsE) as a highly sensitive electrochemical sensing electrode to determine As(III) in water. Figure 1 illustrates the whole picture of this work.

## 2. Materials and Methods

### 2.1. Fabrication of Microporous Graphene

The GF was fabricated by chemical vapor deposition (CVD), where Ni foam was used as the catalyst. C_2_H_2_/H_2_ (3/24) mixtures were flowed through the vacuum tube furnace (0.2 Torr pressure) at 700 °C for 3 min. The tube temperature then underwent rapid cooling (−10 °C/min) with hydrogen flow under 1 Torr pressure, and a graphene layer was formed on the 3D microporous surface. Carbon atoms were dissolved on the Ni Foam scaffold surface, forming graphene/Ni foam. Ni was etched using a 3 M HCl solution at 60 °C for 30 min.

### 2.2. Chemicals and Reagents

Sodium borohydride (NaBH_4_) with 97% purity was purchased from Loba Chemie Pvt. Ltd., Mumbai, India, and iron sulfate heptahydrate (FeSO_4_·7H_2_O) was obtained from Quality Reagent Chemicals (QReC), Wellington, New Zealand. All solutions were freshly prepared with deionized water.

### 2.3. Nanoflower Decoration

The active electrode materials of the sensor were GF and metal-organic nanoflowers. The nanoflower decoration was initiated by diluting FeSO_4_·7H_2_O in a 2 mM NaBH_4_ solution. Afterward, GF was placed in a mixture of FeSO_4_ and NaBH_4_ solution on top of a magnetic stirrer. The mass ratio of FeSO_4_ : GF was maintained at 10:100 for 30 min at room temperature. The grown nanoflowers were characterized by a Field-Emission Scanning Electron Microscope (FESEM).

### 2.4. Material Characterization

**Figure 2:**
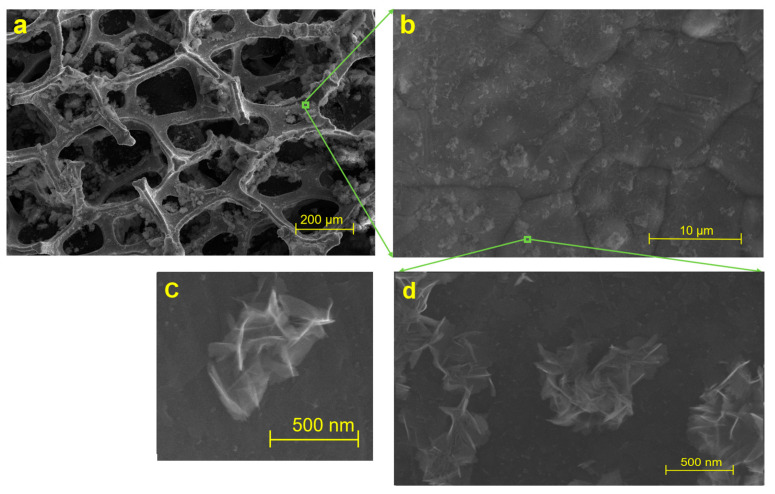
Images showing NF-decorated GF: (**a**) 1000× microscope, (**b**) Field-Emission Scanning Electron Microscope (FE-SEM), (**c**,**d**) FE-SEM showing presence of nanoflowers.

**Figure 3:**
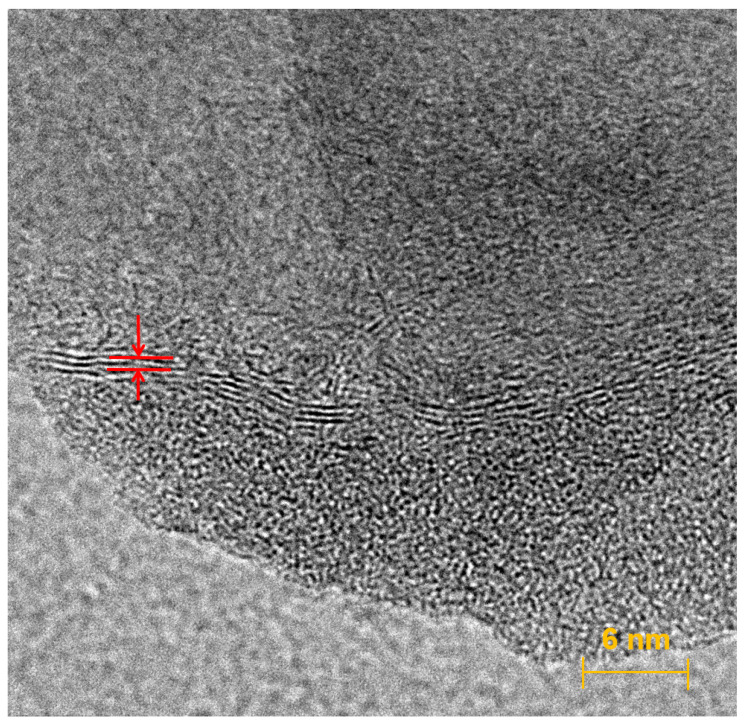
HR-TEM at 500,000 magnification scale. The presence of graphene layers is indicated by red arrows.

The sensor material was characterized by FE-SEM in Figure 2 and High Resolution-Transmission Electron Microscopy (HR-TEM) in Figure 3. The appearance of nanoflowers was observed using FE-SEM. The presence of graphene layers was characterized by HR-TEM at a 500 k magnification scale. For electrochemical measurements, cyclic voltammetry (CV) of the GF/GNFs electrode was performed using different concentrations of As(III) solution, from 50 ppb to 1 ppb. The acquired CV showed a redox potential of 0.15 V. This potential was selected for further investigation of the analytical performance using chronoamperometry. In the experiment, the arsenic solution was dropped at different time points, and the response current was recorded. The difference in the current before and after each drop (1 ppb) was plotted against the concentrations. The measurements were recorded only after the analyte was saturated and became homogeneously distributed in the solution with a magnetic stirrer. In the final set of experiments, the selectivity of the GF/GNFs electrode was investigated using Cd(II), Pb(II), and Cu(II) ion interference. The concentrations and volumes of Cd(II), Pb(II), and Cu(II) droppings were equal to 1 ppm and 10 μL, respectively. The final concentration in a 5 mL solution of the ions was 2 ppb for each drop.

### 2.5. Overall Process

The whole experiment is summarized in Figure 1. The process started with CVD to fabricate microporous graphene foam, the addition of ferrous ions to form metal-organic nanoflowers as ferrous-carbon composites holding negative charges (Fe : G)^−3^, and the detection process. The negative charges (−3*e*) can be described according to ferrous ions and C valence properties and will be explained in the mechanism section. The redox process occurred at the GF/GNF electrode, causing a measurable current proportional to As(III) concentrations. *p* denotes holes after losing electrons from the electrode material, causing a positive current.

## 3. Results and Discussion

### 3.1. Structure of Graphene Nanoflowers or Fractal Micro-Nanoporous Graphene

The surface morphology of GF after the addition of ferrous nanoparticles and a flower-folded internal structure, as characterized by FE-SEM, are illustrated in Figure 2. Metal-organic graphene nanoflowers were well decorated on a three-dimensional GF surface. The microporous structure of GF appeared to be stable (Figure 2a) after the deposition of hybrid metalloorganic nanoflowers (Figure 2b–d). The presence of graphene layers was characterized by HR-TEM, as shown in Figure 3.

### 3.2. Electrochemical Measurements

**Figure 4:**
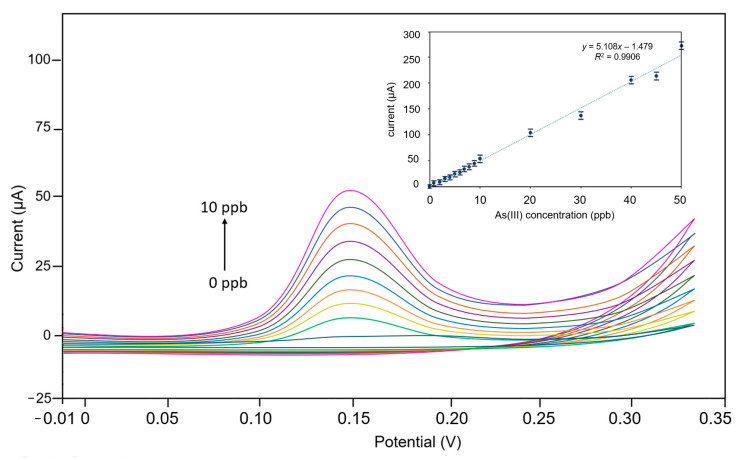
Cyclic voltammogram of GF/GNFs sensors in response to different concentrations of As(III). A smaller frame illustrates the linear relationship between As(III) concentration vs. redox current plot.

The active electrode used was GF/GNFs, whereas Pt-wire and Ag/AgCl electrodes were used as the counter and reference electrodes, respectively. When a cyclic voltammetry (CV) of the GF/GNFs electrode was plotted in response to different concentrations of As(III) ranging from 1 ppb to 10 ppm, the sensor system showed a linear relationship between the redox current and As(III) concentrations (Figure 4). HNO3 (0.1 M) was used as the electrolyte since it yields the highest electrical signal for As(III) compared to the other electrolytes, such as HCl and H_2_SO_4_ [19]. The CV potential was swept from 0–0.3 V, covering the oxidation peak. The peak current was recorded at 0.15 V. This potential was selected for use in chronoamperometry.

**Figure 5:**
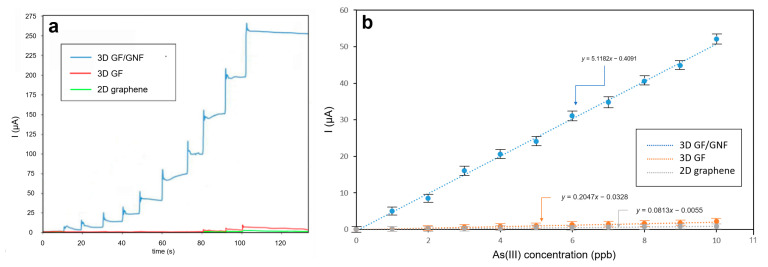
Amperometric responses: (**a**) chronoamperometry comparing (blue) 3D microporous graphene with nanoflowers, (green) bare 3D microporous graphene and (red) flat graphene electrodes in response to drops of increasing concentration of As(III) from 0, 1, 2, …, and 10 ppb arsenic at ordered times, (**b**) linear plot corresponding to Figure 5a.

Chronoamperometric performance analysis of the sensor showed that the measured current was proportional to the arsenic concentration. When the arsenic solution was dropped into the experimental setup, it resulted in a step current (Figure 5). A drop of 1 ppb yielded an increment of 5 μA current, and therefore, the As(III) detection sensitivity of the sensor was 5 μA/ppb or 5000 μA/ppm. Three-dimensional microporous graphene with nanoflowers yielded the highest sensitivity compared to bare microporous graphene and flat graphene (Figure 5a,b). Sensitivity was illustrated by the responses (Figure 5a) and their slopes (Figure 5b). The experiments were repeated to collect the standard deviations (SD), where the current’s SD = 1.51 μA. These yield a computation for LOD = 3.3·SD_y-axis_/slope = 3.3 · 1.51 (μA)/5 (μA/ppb) = 1 ppb.

### 3.3. Interference Test

**Figure 6:**
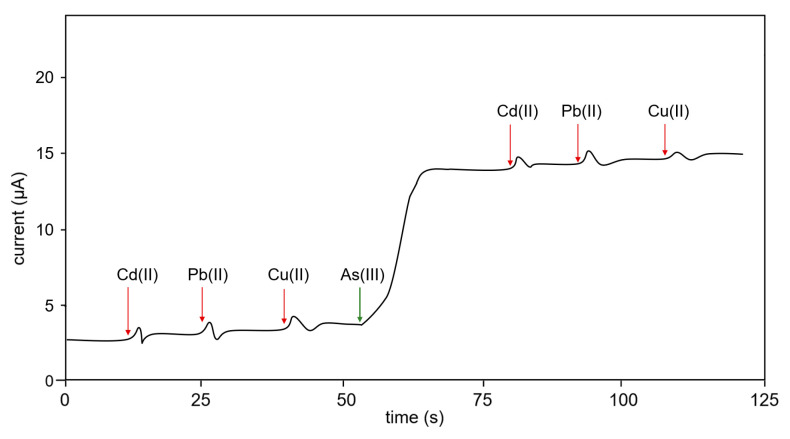
Selectivity test of the sensor. The plot filtered out the noise to identify the substance addition response. Amperometric detection in response to Cd(II), Pb(II), and Cu(II) showed insignificant interference. Equal concentrations of Cd(II), Pb(II), Cu(II), and As(III) at 1 ppm and 10 μL were used in each drop of the experiment (5 mL solution).

The current detected by the sensor electrode in response to other heavy metals, such as Cd(II) and Pb(II), was negligible (Figure 6). The intensity of current recorded by the GF/GNFs electrode on exposure to the heavy elements was comparatively lower than arsenic at various time points. For testing, 1 ppm and 10 μL of Cu(II) were dropped into a 5 mL solution. However, it does not interfere because its valent electron does not match with the composite structure. The electrodes are metal-organic materials that do not form the alloy with the Cu(II).

### 3.4. Electrochemical Impedance Spectroscopy

**Figure 7:**
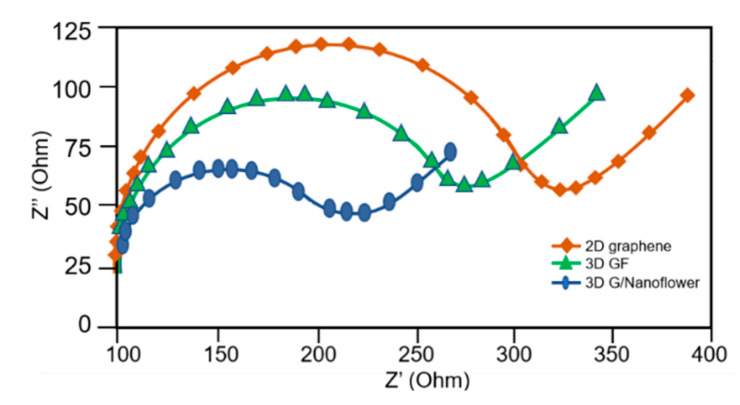
Nyquist plot illustrating electron transfer resistance as the radii. The 3D microporous graphene with nanoflowers showed the lowest impedance.

The behavior of the system can be analyzed by Electrochemical Impedance Spectroscopy (EIS). Usually, the EIS Nyquist plot is used to illustrate the impedance characteristics. Nyquist plots for each case are shown in Figure 7. Together, the electrolyte and electrode form a capacitive structure. It can be written as a double-layer capacitor (*C_dl_*). Its equivalent circuit is composed of a capacitor and charge transfer resistance (*R_ct_*). The radii in the Nyquist plot correspond to the charge transfer resistance of each material. The two-layer fractal microporous graphene proposed in this work showed the lowest impedance compared to flat graphene (2D) and bare microporous graphene. This implies the claim that microporous graphene decorated with a nanoflower structure yields the highest sensitivity among the three methods.

### 3.5. Sensing Mechanism

The mechanism of high sensitivity can be described in two aspects. First, there is a two-level reactive surface area due to the 3D microporous graphene layer and the nanoflowers. These two magnificently increase the surface area in comparison with a flat graphene electrode. Second, the interlayer dipole-dipole enhancement greatly catalyzes the interaction at the electrode of the G-Ni foam [20]. Dipole enhancement and micro-nano surface enrichment synergize to improve the sensing capability of the materials. In addition, ferrous addition was reported to catalyze arsenic binding [21].

**Figure 8:**
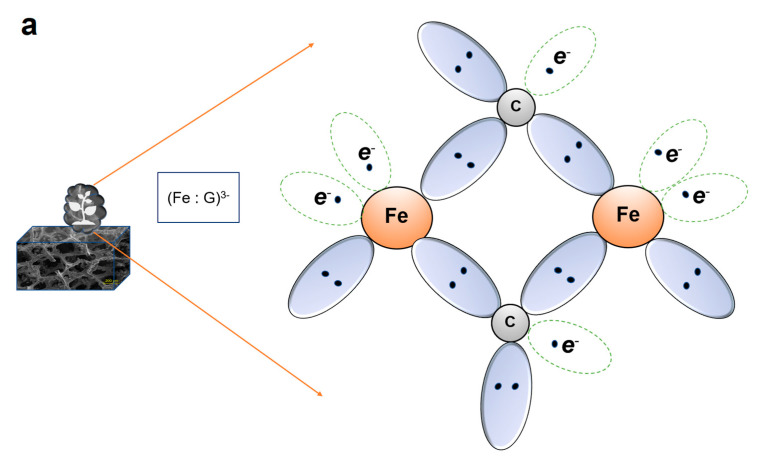

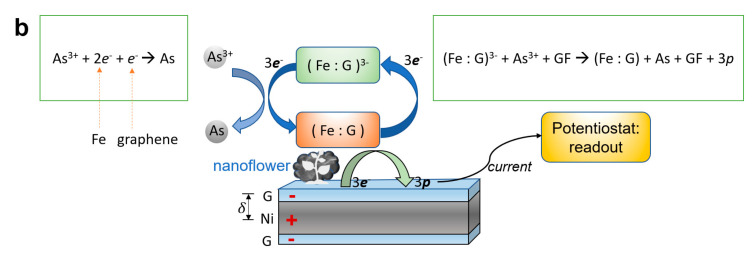
Mechanism (**a**) Fe_2_O_3_ flowers synthesis (**b**) sensing mechanism.

For specificity aspects, the hybrid carbon-ferrous nanoflowers (Fe: G)^3+^ greatly support the selectivity for As(III) rather than As(V) (Figure 8). GF/GNFs could be selective and highly sensitive As(III) sensors, as illustrated in Figure 8. Arsenite (As^3+^), once dissolved in water and moving near the electrode, is reduced into As^0^, resulting in a reduction current of 3*e* at the working electrode. Each As^3+^ ion gains 2 free electrons from Fe and a free electron from graphene, where they formed as graphene-ferrous composites (Fe : G)^3−^ in the shape of nanoflowers.

### 3.6. Reproducibility

**Figure 9:**
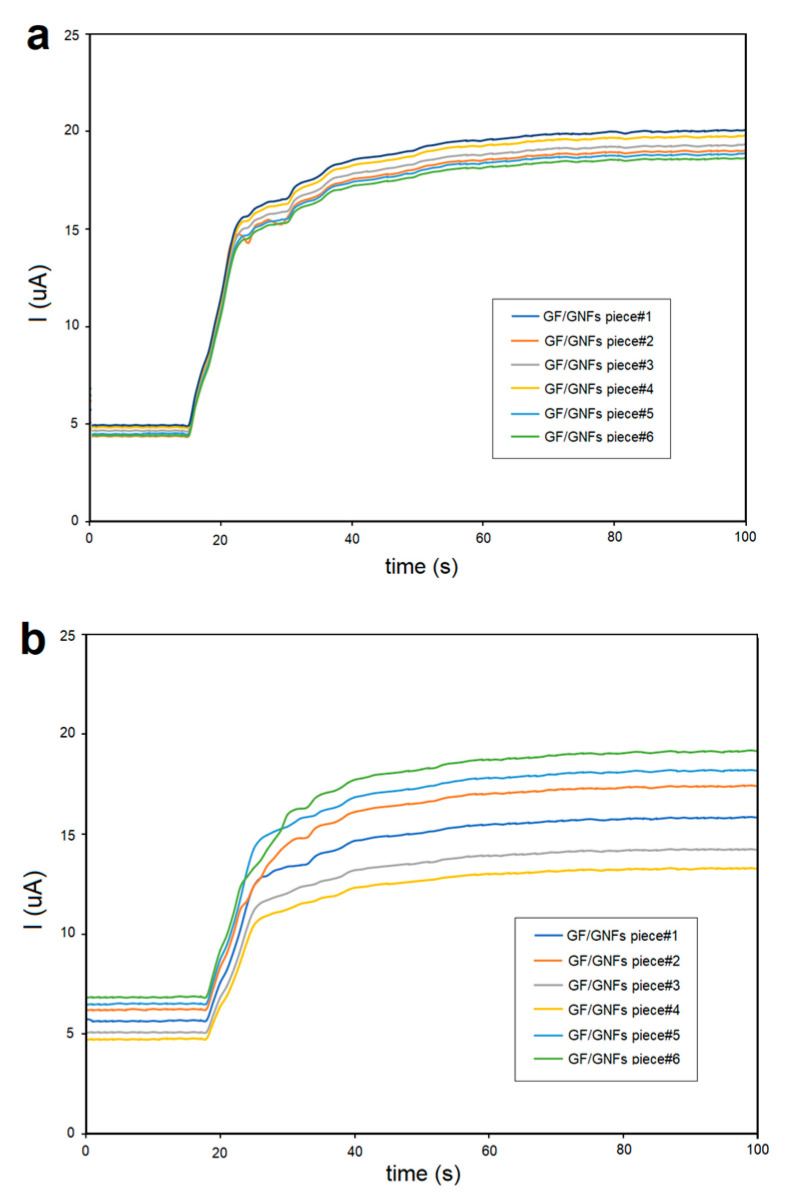
Reproducibility using 6 different pieces of GF/GNFs electrode and repeat the chronoamperometry: (**a**) day 1 yields RSD% = 6, (**b**) 7 days passed RSD% lift to 10. Note that samples #1,#2,…,#6 were synthesized using equivalent conditions.

For reproducibility testing, we prepared 6 different pieces of GF/GNFs electrodes where the same amperometric measurement was carried out. The results yielded RSD% = 6 on day 1 and kept increasing until RSD% reached 10 on day 7 (Figure 9). It thus concludes that the proposed electrode was reproducible and reusable for up to 7 days with once-a-day use. The working electrode was cleaned with water rinsing before the next repetition test. Meanwhile, Ag/AgCl was kept in a 3 M NaCl solution during the interval time, and the Pt reference electrode was cleaned with 10% nitric acid in deionized water and submerged for 5 min before the next usage.

### 3.7. Actual Tests on Environmental Water Sources

We sampled 5 natural water sources where the reference value of As(III) was evaluated by the official authority using the standard ICP-MS method. The results are shown in Table 1. The mean absolute error was 0.184 ppb, which is insignificant compared to the scale of the US EPA cut-off value of 10 ppb.

### 3.8. Discussion

Nanoflower-based microporous graphene was fabricated successfully using Chemical Vapor Deposition (CVD). A ferrous layer on graphene was formed and deposited by iron (ii) sulfate and sodium borohydride as precursors for reactions. The mass ratio between FeSO_4_ and GF was kept at 1:10 in the experiment. As a result, the structure determined by FE-SEM appeared to be metal-organic nanoflowers folded on the microporous graphene surface. These nanoflowers greatly enhance the reactive surface area of the working electrode. Graphene nanoflowers give a hierarchical structure to the graphene foam surface. This nanostructure could be said to be fractal, literally. According to several articles relating to metalloorganic structures, it normally yields a flower-like structure [18]. In our case, the mechanism involved reducing ferrous compounds by sodium borohydride (NaBH_4_) on the graphene surface and forming metalloorganic compounds. Iron (ferrous) has been reported to catalyze the reaction to As(III) [21]. Therefore, it was deposited into microporous graphene to further improve sensitivity and selectivity in the study.

To determine the potential for amperometric detection, we carried out cyclic voltammetry with varying As(III) concentrations from 1–50 ppb. The redox potential was found in the cyclic voltammogram at 0.15 V. This potential was selected for chronoamperometry. In Figure 5, we compared the sensitivity of using three different active electrodes: (i) nanoflowers/microporous graphene, (ii) microporous graphene foam, and (iii) flat graphene. The results of the study showed that the graphene nanoflower electrode has significantly higher sensitivity compared to ordinary graphene foam and flat graphene, with an improvement of 50–60 times.

For the selectivity test, Cd(II), Pb(II), and Cu(II) solutions were dropped onto the test solution and gave an insignificant current. Thus, the specificity of this sensor to As(III) can be concluded against at least two sources of interference. Further tests can also be conducted; however, the reaction is specific to the redox potential of our focused analyte, i.e., As(III).

Electrochemical impedance spectroscopy was carried out, and the Nyquist plots are included in Figure 7. Charge transfer resistance was lowest in graphene nanoflowers and nano-microporous structures. This was in accordance with chronoamperometry, where graphene nanoflowers showed the highest sensitivity among the three types of electrodes.

A comparison between the other methods and our developed nanoflower-based nano-microporous graphene sensor for arsenic measurements is shown in Table 2. We emphasize that the sensor is a promising method for arsenic determination in water.

The key findings in this work are listed below.

- Novel nanoflowers grown on a microporous graphene foam surface formed a gigantic surface area enrichment and a highly sensitive electrode.
- LOD achieved was 1 ppb, comparable to lab-scale methods, such as ICP-MS, HPLC, and AAS.
- Sensitivity was measured as 5000 μA/mgL^−1^ (response current/As(III) concentration), which was 50–60 times greater than that of bare microporous graphene and flat graphene.
- It was highly specific to As(III) under Cd(II), Pb(II), and Cu(II) interference.
- It had a low cost compared to the AuNPs-based method and other large laboratory-scale methods.
- Portable devices and on-site investigation of As(III) contamination are possible for the offensive prevention of As(III) contamination in agricultural water and drinking water.
- It required a smaller sample size in comparison with other spectroscopic-based approaches, such as AAS, ICP MS, and AFS.
- Amperometric detection supports rapid readout and is suitable for field experiments for actual As(III) screening.
- Reproducibility was tested, and RSD% gradually increased from 6% to 10% within 1 week.

The reproducibility of the proposed nanoflowers/GF electrode was tested using a 20-time repeated experiment, and the standard deviation (SD) of each method is reported in Table 3.

## 4. Conclusions

A highly sensitive electrochemical sensor based on metal-organic nanoflowers on microporous graphene was developed and tested for As(III) sensing in water. This method yields the greatest sensitivity in comparison with flat graphene and microporous graphene. An explanation of greater sensitivity could be the larger reactive surface area due to nanoflower structure folding on a microporous graphene foam surface. In addition, a catalytic mechanism due to dipole-dipole enhancement between graphene and the nickel layer within the porous further increases the electron transfer [20]. The proposed method is feasible for composing a low-cost, highly sensitive electrode sensor with easy integration into miniaturized devices. In the study, the ultra-high sensitivity achieved by the sensor could be explained by its extraordinarily large reactive surface area and the nanoflower folding on the GF. Moreover, the use of iron in our sensor has been reported to increase electrode interaction with arsenic [21]. Nonetheless, it was the first instance where graphene nanoflower-based electrodes were shown to have a great sensitivity for arsenic detection. The fabricated materials possessed a two-step signal enhancement mechanism. The first is from microporous graphene, and the second is from nanoflowers. Ferrous was deposited onto the nanoflowers on microporous graphene to further catalyze electrode binding with As(III). The current sensor system was capable of detecting trace amounts of As(III) in water. Moreover, the sensor’s working range covers the cut-off level of arsenic at 10 ppb, as given by the US EPA. The selectivity achieved was possibly due to the potential selection according to the CV peaks and catalytic binding to As(III) via ferrous addition. In short, features such as sensitivity, selectivity, simplicity, reproducibility, and portability of the newly developed sensor make it a promising candidate for screening arsenic levels in consumption and environmental water. This invention could be vital in preventing arsenic toxicity in humans. The proposed method could be extended worldwide to probe arsenic contamination in drinking water and water in natural resources. This would contribute to global health by providing safe water in any part of the world.

## Figures and Tables

**Table 1:** Comparison–experimental results on 5 natural water resources’ As(III) contamination determined by our method and the reference (ICP-MS) method.

Natural Water Resource Label	1	2	3	4	5
our method	5.0 (ppb)	3.0 (ppb)	4.0 (ppb)	5 (ppb)	7 (ppb)
reference method	5.21 (ppb)	2.78 (ppb)	4.15 (ppb)	4.86 (ppb)	7.20 (ppb)

**Table 2:** Comparison of the methods for arsenic measurement.

Materials/ Methods	Detected Species	LOD (μg/L)	LR (μg/L)	Advantages	Disadvantages	Ref.
GO nanosheet	As(III)	500	not reported	Carbon-based: low toxicity and low cost	low sensitivity	[3]
HPLC-ICP-MS	Total arsenic and arsenic speciation	0.6–6	0.005–10	US EPA approved	expense	[6]
MSPE with HPLC-ICP-MS	As(III)	0.0011	1–10	ultra-high sensitivity	Expensive and not real-time available on site	[6]
AuNPs + colorimetry	As(III)	2.0	5–500	On-site detection	more expensive than carbon-based method	[8]
Pt/Au nanoparticles + PANI	As(III)	1.48	2.47–14.98	ease of use	Expenses of Au and Pt precursor	[11]
Fe_3_O_4_–rGO/SWV	As(III)	1.19	1–20	Carbon-based: low toxicity and low cost	moderate sensitivity	[12]
porous gold electrode	As(III)	0.1	0.1–70	high sensitivity	expense in Au nanostructure fabrication	[13]
Fluorescent	As(III)	0.18	0.5–2.99	moderate sensitivity	expensive, dilution required before measurement	[22]
ICP-MS	Total arsenic	~0.1	Tunable	US EPA approved	Expensive and Spectral interference	[23]
Graphene-Pt	As(III)	0.008	0.75–7.5	ultra-high sensitivity	cost and time to synthesize graphene-platinum composites and required dilution before measurement	[24]
GF + graphene nanoflowers	As(III)	1.0	1–50	lower cost, rapid readout and on-site availability	not reported	This work

**Table 3:** Reproducibility of the proposed method.

Measurement Method	Value	SD
CV	I (μA)	1.2 μA
Chronoamperometry	I (μA)	1.51 μA
EIS	R_ct_ (Ohm)	4.1 Ohm

## Data Availability

Data are contained within the article.
